# Biotic Interactions Shape Soil Bacterial Beta Diversity Patterns along an Altitudinal Gradient during Invasion

**DOI:** 10.3390/microorganisms12101972

**Published:** 2024-09-28

**Authors:** Yuchao Wang, Wenyan Xue, Jinlin Lyu, Ming Yue, Zhuxin Mao, Xuejian Shen, Xue Wang, Yang Li, Qian Li

**Affiliations:** 1 Xi’an Botanical Garden of Shaanxi Province (Institute of Botany of Shaanxi Province), Xi’an 710061, China; 2 Shaanxi Engineering Research Centre for Conservation and Utilization of Botanical Resources, Xi’an 710061, China; 3 Xi’an Ecological Monitoring and Restoration Engineering Technology Research Center, Xi’an 710061, China; 4 Shaanxi Key Laboratory of Qinling Ecological Security, Shaanxi Institute of Zoology, Xi’an 710061, China; 5 School of Life Sciences, Northwest University, Xi’an 710069, China; 6 Forest Disease and Pest Control and Quarantine Station of Shangluo, Shangluo 726000, China

**Keywords:** invasive plants, beta diversity partition, distance–decay relationships, iCAMP model, co-occurrence patterns

## Abstract

Invasive plants have already been observed in the understory of mountain forests, which are often considered a safe shelter for most native plants. Microorganisms might be drivers of plant invasions. Nevertheless, the mechanisms determining variations in microbial community composition (beta diversity) during invasion along altitudinal gradients remain to be elucidated. Here, the elevational patterns and the driving ecological processes (e.g., environmental filtering, co-occurrence patterns, and community assembly processes) of soil bacterial beta diversity were compared between invasive and native plants on the Qinling Mountains. The species turnover dominated bacterial compositional dissimilarities in both invasive and native communities, and its contribution to total beta diversity decreased during invasion. Total soil bacterial dissimilarities and turnover exhibited significant binominal patterns over an altitudinal gradient, with a tipping point of 1413 m. Further analysis showed that the contributions of assembly processes decreased in parallel with an increase in contributions of co-occurrence patterns during the invasion process, indicating that species interdependence rather than niche partitioning is strongly correlated with the bacterial biogeography of invasive communities. Plant invasion affects the relative contributions of stochastic processes and co-occurrence interactions through the regulation of the physiochemical characteristics of soil, and ultimately determines compositional dissimilarities and the components of the bacterial community along altitudinal gradients.

## 1. Introduction

The Qinling Mountains represent the most significant geographical–ecological transition zone in China, playing indispensable roles in maintaining biodiversity and ecosystem functionality with its high species richness and distinctive species composition [1,2]. Nonetheless, the mountain ecosystems have been subjected to considerable pressure from a rapidly expanding population, land-use changes (such as agricultural expansion, urbanization, and other infrastructure developments), and the excessive exploitation and irrational utilization of forest resources in recent years. A prominent consequence of intensified anthropogenic interference is its facilitation of the introduction and settlement of invasive species in mountains, gradually depriving shelters for the survival of native species by altering the understory of mountain forests [3]. Soil microbes could be either responsive to or primary drivers of plant invasions, and one fundamental goal of invasion studies is, therefore, to elucidate how the diversity of soil microorganisms responds to invasion [4]. Previous research has primarily focused on coexisting species numbers in microbial communities (α diversity) during invasion [5,6]. However, there is still a paucity of knowledge regarding community compositional dissimilarity (beta diversity), including turnover (replacement of species) and nestedness difference (loss or gain of species), in response to invasion. As a consequence of global climate change and the increasing time since colonization, invasive plants may extend their distribution ranges and reach new areas in the future [7]. A comparison of beta diversity patterns of rhizosphere soil microbes from native and invasive plants along elevation gradients, coupled with an investigation of the causal factors behind these patterns, could provide insights into the mechanisms underlying the invasion processes of alien plants.

The growing interest in beta diversity is driven by the recognition of its crucial roles in revealing community assembly mechanisms [8], among which the debate about whether community composition is driven by deterministic and stochastic processes has attracted more and more attention [9,10]. The deterministic processes refer to niche-based processes such as environmental selection, while birth/death, speciation extinction, mutation, and immigration (or dispersal) constitute the stochastic processes. Previous studies have demonstrated that the microenvironment of soil microorganisms, including soil pH and nutrient availabilities, are significantly altered during invasion [11], which exerts selective forces on microbial community selection, leading to a higher (heterogeneous selection) or lower (homogeneous selection) community divergence [12]. However, Zhang et al. [4] reported that patterns of bacterial beta diversity were primarily driven by dispersal limitation and selection along the invasion gradient, whereas Bell et al. [13] observed minimal effects of invasion on the assembly process. One potential explanation for the inconsistent findings is that contributions of ecological processes were inferred from the overall analysis of the microbial community in these studies, ignoring the subcommunity assembly mechanisms of microbial lineages at fine taxonomic levels, which has been reported to be highly variable [14,15]. For instance, homogenous selection was responsible for 70% of the assemblage of Crenarchaeota, whereas it accounted for approximately 10% of the assemblage of Actinomycetes, Gemmatimonadetes, and Planctomycetes [15]. Recently, the Infer Community Assembly Mechanisms (iCAMP) analysis was developed to determine the microbial subcommunity assembly mechanism of functional groups at bin levels. Based on this method, the assembly mechanisms of soil microbial subcommunities at finer taxonomic levels and their determining factors have been explored in a variety of ecosystems, such as forests [16], grasslands [17], and farmlands [18]. However, our understanding of the balance between deterministic and stochastic processes in microbial subcommunities assemblage under plant invasion is extremely limited.

An alternative deterministic process, biotic interactions, illustrates the impact of biological selection on microbial beta diversity [19]. That is, what differentiates a community from a random set of taxa is precisely these interactions, as they give rise to properties at the community level that cannot be understood by considering each taxon in isolation [20]. Theoretically, weaker biotic interactions (high inter- and low intra-specific competition) may result in an increase in the abundance of the same species and the exclusion of other species, which would lead to a reduction in beta diversity. Conversely, lower interspecific competition may create niches for other species and limit similarity, ultimately leading to high beta diversity [19]. Co-occurrence network analysis is a frequently employed method for inferring potential microbial interactions within given habitats, reflecting fluctuations in microbial abundance patterns in response to a common environmental factor. Previous researchers have identified altered co-occurrence networks of soil microbes resulting from variations in microbial diversity and composition during invasion [21,22]. Nevertheless, the extent to which biotic interactions influence the beta diversity of soil microorganisms remains uncertain, particularly with regard to its turnover and nestedness components, which impedes a more robust understanding of the relationship between spatiotemporal diversity patterns and community composition. Additionally, the relative contributions of co-occurrence patterns to the diversity and composition of microbial communities remain poorly understood in comparison to community assembly processes.

Previously, we have conducted a comprehensive assessment on the invasive risks of non-native plant species in the Qinling Mountains, with the results indicating that *Conyza canadensis*, *Erigeron annuus*, and *Galinsoga quadriradiata* exhibited the highest risk of invasion [23]. Of these three invasive plant species, *Galinsoga quadriradiata* has the shortest period of colonization, and thus is the least understood invasion process. Here, rhizosphere soil samples were collected from *Galinsoga quadriradiata* along an elevational gradient (elevation gradient from 896 to 1889, consists of six elevations) on the Qinling Mountains, taking *Artemisia lavandulaefolia* as a native control, to demonstrate the altitude patterns of bacterial beta diversity and their potential mechanisms. Firstly, the total community compositional dissimilarities were partitioned into turnover and nestedness-resultant components, to explore whether and in what ways the bacterial beta diversity of invasive and native communities exhibited differentiation. Then, we assessed bacterial beta diversity patterns in relation to elevational and environmental distance. Finally, we examined the relative contribution of environmental selection, dispersal limitation, ecological drift, and biotic interactions in driving beta diversity patterns by applying the iCAMP models and co-occurrence networks. The following hypotheses were tested through these efforts: (i) soil bacterial community similarity significantly declined with elevation distances, and the distance–decay relationships were more pronounced for invasive plants; (ii) deterministic processes dominated the bacterial assemblage of invasive plants, due to the heightened selective forces on microbial communities; (iii) invasion altered the relative importance of ecological processes and co-occurrence patterns to bacterial beta diversity and its components.

## 2. Materials and Methods

### 2.1. Study Area

This study was carried out at Huanghualing (108°52′56.9″ N and 33°47′44.6″ E), a 2328 m-high ridge within the Niubeiliang National Nature Reserve in eastern Zhashui County, Shaanxi Province, situated 31 km uphill from the Qinling Watershed. The average annual temperature and annual precipitation were 2–10 °C and 850–950 mm, respectively, with a frost-free period of approximately 130 days.

The spatial distributions of vegetation types exhibit a distinct vertical zonal pattern along the altitudinal gradient. At an altitude of 2000 m or less, the dominant vegetation type is pine–oak mixed forests, with *Pinus armandii* and *Quercus aliena* as the foundation species, and the dominant tree species being *P. tabuliformis*, *Q. variabilis*, *Q. wutaishanica*, and *Populus davidiana*. The understory shrubs are primarily Lespedeza bicolor, *Spiraea salicifolia*, and Lonicera japonica, while the dominant understorey herbs include *Carex tristachya*, *Deyeuxia arundinacea*, *Dendranthema indicum*, and *Pyrola calliantha*. At altitudes exceeding 2000 m, the predominant vegetation type is birch forest, with *Betula albosinensis* representing the most abundant species, followed by *B. luminifera* and other trees such as *P. armandii*, *Tilia tuan*, and *Abies fabri*. Shrubs in the understory include *Corylus heterophylla*, *Spiraea salicifolia*, and *Sorbus pohuashanensis*; herb species identified within the forest environment include *C. tristachya*, *Oxalis corniculate*.

### 2.2. Rhizosphere Soil Sampling

Soil samples were collected from three randomly selected individuals of *Galinsoga quadriradiata* (invasive plant) and *Artemisia lavandulaefolia* (native plant) each at six elevations (896, 1056, 1202, 1413, 1713, and 1889 m) in Aug 2022. All the selected individual plants were dug up and the bulk soil was shaken off. Then, a sterile soft-bristled brush was used to strip away the rhizosphere soil that was tightly adhering to the surface of the root system. Ultimately, three soil samples were obtained for each elevation per species, resulting in a total of 72 samples (6 elevations × 4 species × 3 replicates).

The collected soil samples were transferred on ice immediately to the laboratory in the Xi’an botanic garden for pre-processing. Each sample was divided into two parts, one air-dried at room temperature for the determination of soil physiochemical properties, and the other stored at −80 °C for the extraction of soil DNA. The concentration of soil organic carbon (SOC), total nitrogen (TN), and phosphorus (TP) was quantified using the K_2_Cr_2_O_7_ oxidation, Kjeldhal, and Mo-Sb anti-spectrophotography methods, respectively. Soil pH was quantified by the potentiometric method.

### 2.3. Soil DNA Extraction and Sequencing

The E.Z.N.A. ^®^ Soil DNA Kit (Omega Bio-Tek, Norcross, GA, USA) was used to extract total genomic DNA from 0.5 g of soil samples, and DNA concentration and purity were assessed using a NanoDrop 2000 spectrophotometer (Thermo Scientific, Wilmington, DE, USA). The primer pairs 338F (5′-ACTCCTACGGGAGGCAGCAG-3′) and 806R (5′-GGACTACHVGGGGTWTCTAAT-3′) were used to amplify the V3–V4 region of the soil bacterial 16 S rRNA gene. Sequencing was conducted on an Illumina HiSeq. 2500 v4 platform (Illumina Inc., San Diego, CA, USA) at Shanghai Majorbio Bio-Pharm Technology Co., Ltd., Shanghai, China.

The resulting sequences were processed using USEARCH v11 to merge paired-end sequences, eliminate primer sequences, and filter out low-quantity sequences (quality score < 20, containing ambiguous nucleotides or not matching the prime). The filtered sequences were then clustered into operational taxonomic units (OTUs) with a similarity threshold of 97% using UPARSE. Representative sequences were selected and annotated with taxonomic information for each OTU using the Silva138 database. Prior to diversity analysis, the OTU tables were rarefied according to the minimum number of sequences to ensure homogeneity of the sample sequenced, resulting in a final sum of sequence abundance of 26,939 for each sample. All sequencing data can be found at the Sequence Read Archive (SRA) of the National Center for Biotechnology Information (NCBI, Bethesda, MD, USA) under BioProject number PRJNA1152501 (https://dataview.ncbi.nlm.nih.gov/object/PRJNA1152501 (accessed on 17 September 2024)).

### 2.4. iCAMP Analysis of Community Assembly Processes

The iCAMP analysis was performed to quantitatively estimate the influences of stochastic (ecological drift, dispersal limitation, and homogenizing dispersal) and deterministic (heterogeneous selection, and homogeneous selection) processes on variations in the beta diversity of soil bacterial communities. This was achieved through the following steps.

First, bacterial OTUs were divided into bins according to their phylogenetic relationships, with altitude used to determine the phylogenetic signal. This resulted in a bin size and threshold of phylogenetic distance of 48 and 0.025, respectively. Secondly, the relative contributions of community assembly processes within each bin were evaluated using the betaNRI (beta nearest relatedness index). |betaNRI| > 1.96 indicated that deterministic processes drove communities, while |betaNRI| ≤ 1.96 indicated that stochastic processes were the predominant forces shaping community assembly. betaNRI values of <−1.96 and >1.96 were indicative of the predominance of homogeneous selection and heterogeneous selection, respectively. Thirdly, RCbray (Bray–Curtis-based Raup–Crick metric) was calculated to further identify the stochastic processes. RCbray < −0.95, RCbray ≤ 0.95, and RCbray > 0.95 represented the influences of homogenizing dispersal, ecological drift, and dispersal limitation, respectively. Finally, the relative importance of different ecological processes to community assembly was ascertained based on the relative abundance of each bin.

### 2.5. Co-Occurrence Network Construction 

To provide supporting evidence for linkages between beta diversity and biotic interactions, and to evaluate whether biotic selections were more important for community composition, we investigated the co-occurrence patterns of soil bacterial for invasive and native plants based on Spearman correlation networks. The network was constructed using OTUs with a relative abundance of more than 0.01%, and only significant and robust correlations (|r| > 0.6, *p* < 0.001) retained. Several topological properties, such as nodes numbers, edge numbers, positive edges, and negative edges, were extracted to describe topological characteristics of the overall network.

We also calculated the topology of the sub-networks in each soil sample as the potential biotic factors determined variations in bacteria beta diversity, including average degree (AD), average path distance (APD), Cenbet (betweenness centrality), Cendeg (degree centrality), Ceneig (eigenvector centrality), Den (graph density), and Trans (transitivity). The sub-networks were derived by preserving the phylotypes of individual soil samples using the induced_subgraph function in R package igraph 2.0.3. Networks were visualized using the interactive Gephi platform 0.10.1 (https://gephi.org (accessed on 6 May 2024)).

### 2.6. Statistical Analysis

Compositional dissimilarities between pairwise communities (beta diversity) were calculated using three metrics: BetaSOR (total Sørensen dissimilarity), BetaSIM (replacement components of Sørensen dissimilarity), and BetaSNE (nestedness-resultant components of Sørensen dissimilarity). Differences in soil bacterial beta diversity and its components (with normal distribution) between invasive and native communities were compared by one-way ANOVA followed by Tukey’s multiple comparison test, using the package vegan. Linear or quadratic polynomial models were fitted to examine patterns of beta diversity with altitudinal gradients and environmental distances. The environmental distance was quantified as the standardized Euclidean distance (standard deviation = 1 and mean = 0) for each of the soil physiochemical properties investigated, including soil pH, soil total nutrients (C, N, P, and K), and stoichiometry (C:N, C:P, N:P). The correlations between bacterial beta diversity and edaphic variables/ecological processes/network topological properties were tested using Mantel tests. The independent or combined effects of three groups of variables (edaphic properties, ecological processes, network topological characteristics) on explaining changes in community composition were assessed through variance portioning analysis. To estimate the relative importance of various predictors in the explanation of the community composition matrix, random forest analysis was conducted using the randomForest package. The contribution of each variable was determined by the increase in mean squared error (%IncMSE) calculated by the ‘importance’ function, and the adjusted R-squared and significance of the random forest models were assessed using package A3. All statistical analyses and visualizations were conducted in R 4.3.1.

## 3. Results

### 3.1. Soil Bacterial Beta Diversity Patterns along Altitudinal Gradients 

**Figure 1:**
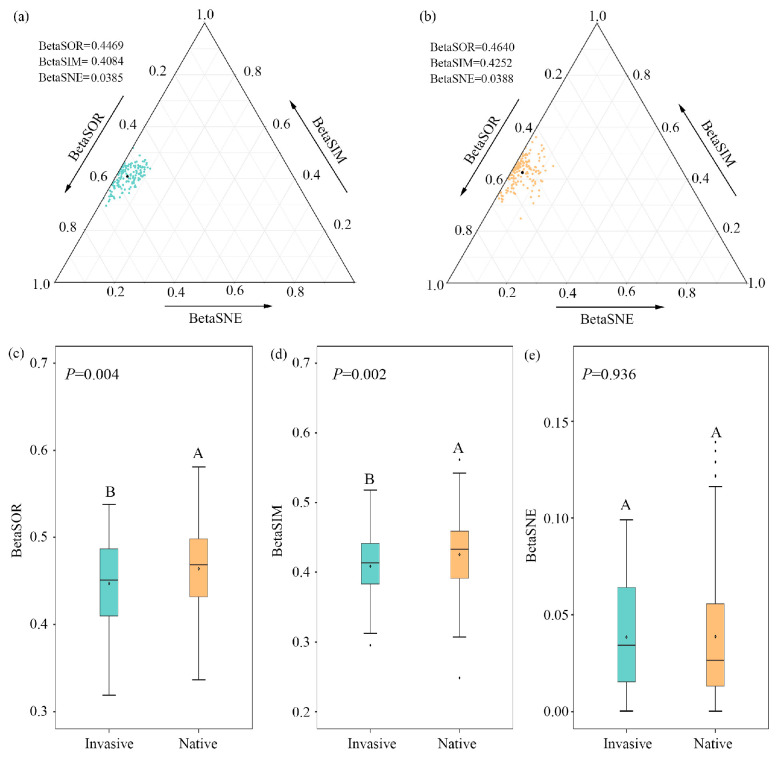
Triangular plots of bacterial beta diversity for (**a**) invasive and (**b**) native plants. Points represent pairs of samples, with position determined by the triplet values in the BetaSOR (total Sørensen dissimilarity), BetaSIM (turnover component of Sørensen dissimilarity), and BetaSNE (nestedness component of Sørensen dissimilarity) matrices. The mean values are shown as black dots. (**c**–**e**) Comparisons of dissimilarities and partitioned turnover and nestedness components for pairwise bacterial communities between native and invasive plants. Different letters represent significant differences of bacterial beta diversity between native and invasive communities.

The decomposition analyses of beta diversity demonstrated that bacterial compositional dissimilarities across altitudinal samples were primarily driven by species replacement for both invasive and native communities, contributing 91.40% and 91.60% on average; nestedness difference only accounted for 8.60% and 8.40%, respectively (Figure 1a,b; Table S1). The total compositional dissimilarity and species replacement decreased significantly during invasion (*p* < 0.01), while no significant (*p*  =  0.936) differences were observed for species loss/gain between invasive and native communities (Figure 1c–e).

**Figure 2:**
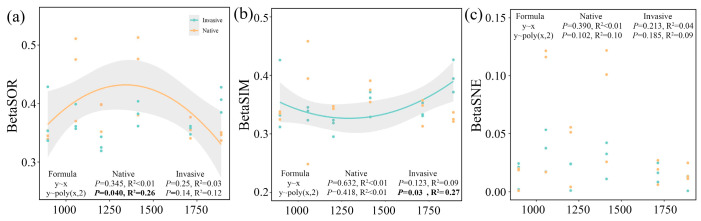
Relationships between altitudinal gradients and total dissimilarities (**a**), replacement (**b**), and nestedness (**c**) component of bacterial community. Linear and binomial regressions were fitted. Solid lines indicate significant relationships (*p* < 0.05). Statistics parameters of significant regression are bolded. The significant fitted regressions are shown in bold.

The beta diversity along elevational gradients displayed no distance–decay relationships (*p* > 0.05), but rather a significant binominal relationship with a tipping point of 1413 m (Figure 2). Notably, the community dissimilarity of native samples exhibited an inverted U-shaped trend over elevational gradients (*p* < 0.05, Figure 2a), whereas a diametrically opposed U-shaped trend was observed for species replacement of invasive bacterial communities (*p* < 0.05, Figure 2b). No valid decay or binomial relationships (*p* > 0.05) were observed between species loss/gain and elevational distances for both native and invasive communities (Figure 2c).

However, beta diversity has a significant decay relationship with the environmental distance (Figures S1–S3). pH distance possessed a higher correlation coefficient than other environmental variables (soil nutrients and stichometry). The effects of environmental distance on species turnover (Figure S2) were much stronger than those on species loss/gain (Figure S3). Furthermore, in contrast to bacteria from native plants, analyses of linkage decay for invasive communities showed a stronger relationship between beta diversity and environmental distance in most cases (Figures S1–S3).

### 3.2. Bacterial Assembly Process Based on iCAMP Models 

**Figure 3:**
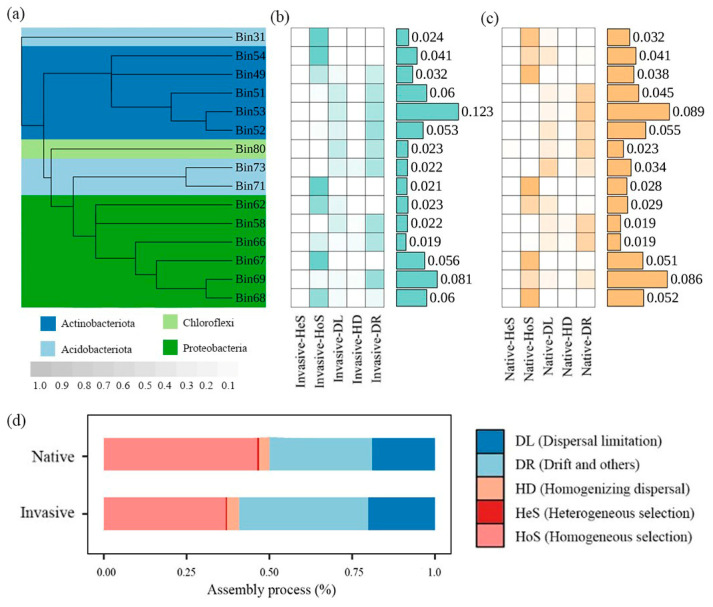
Variations in ecological processes based on iCAMP analysis. (**a**) The phylogenetic tree of the top 15 bins. (**b**) The heatmaps showing major assemblage processes governing dominant bins in invasive bacterial communities with color gradient showing relative importance of each process, and values in bar plots showing relative abundance of each bin. (**c**) The heatmaps showing major assemblage processes governing dominant bins in native bacterial communities with color gradient showing relative importance of each process, and values in bar plots showing relative abundance of each bin. (**d**) Relative contribution of different ecological processes to all bins at community levels.

The iCAMP analysis yielded 91 phylogenetic bins based on phylogenetic relationships of 13,245 OTUs. The number of bins dominated by dispersal limitation decreased while bins dominated by ecological drift increased in the invasive community. Of the 15 OTU bins most strongly affected by assembly processes (64.09–66.04% of relative abundance), seven bins were regulated by homogeneous selection and six were regulated by ecological drift (Figure 3b).

When combining ecological processes among all bins, we found that the assembly of the bacterial community was dominated by stochastic processes, with a greater prevalence in invasive communities (62.79%) compared to native plants (53.06%) (Figure 3a). Drift and homogeneous selection were the most significant contributors to bacterial community assembly along elevational gradients. The bacterial communities of invasive plants exhibited a significantly higher ratio of dispersal limitation and drift, but lower homogeneous selection than those of native communities (Figure 3).

### 3.3. Biotic Interactions Based on Bacterial Co-Occurrence Patterns 

**Figure 4:**
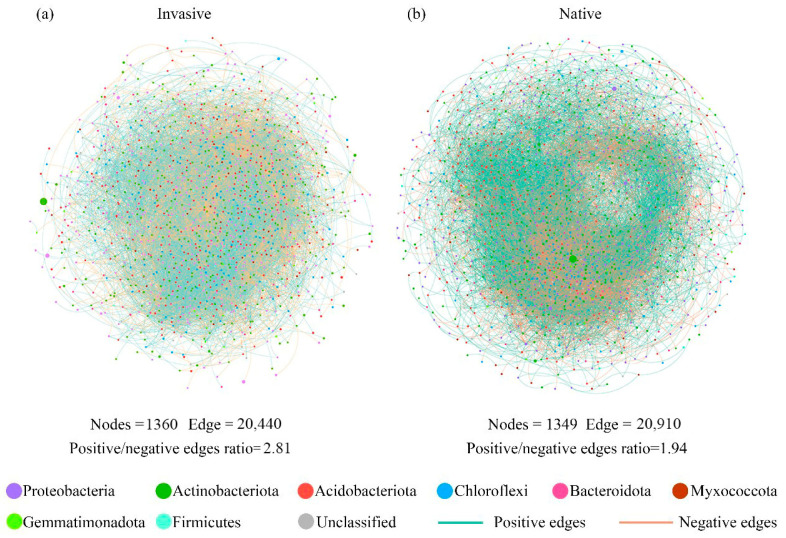
The soil bacterial co-occurrence networks for (**a**) invasive and (**b**) native plants based on correlation analysis. The networks are colored by the bacterial phyla, and the size of each vertex corresponds to their relative abundance. The positive interactions between two nodes are displayed with green edges, while negative interactions are displayed with red lines.

Varying co-occurrence networks of soil bacterial taxa were observed between invasive and native communities. The invasive and native networks captured 20,440 edges among 1360 vertices (Figure 4a), and 20,910 edges among 1349 vertices, respectively (Figure 4b). In addition, the native co-occurrence networks harbored a lower positive/negative edge ratio (1.94) in comparison to the value in invasive communities (2.81) (Figure 4). Since the positive and negative edges of co-occurrence networks may be indicative of species cooperation and competition, the lower positive/negative edge ratio probably indicated stronger species competition in native communities.

The topological parameters of co-occurrence sub-networks, including betweenness centrality, graph density, and network transitivity, were found to be significantly higher in invasive communities compared to those in native communities (Table S2). The average path distance exhibited an increase during invasion, but the difference with native community was statistically insignificant (*p* > 0.05).

### 3.4. Underlying Drivers of Soil Bacterial Beta Diversity along Elevational Gradients 

**Figure 5:**
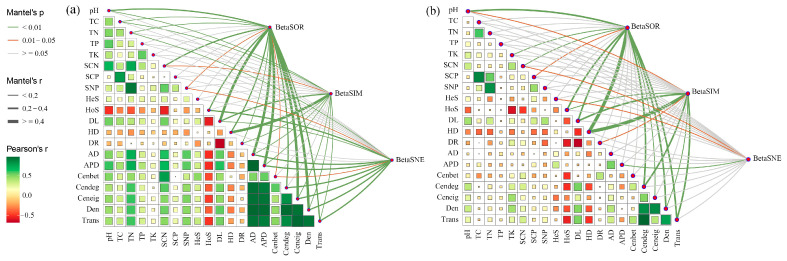
The distance correlations of the bacterial community dissimilarity with edaphic variables, assembly process, and network properties in the rhizosphere of invasive (**a**) and native (**b**) plants determined using the Mantel test. Mantel’s r is proportional to line width, and *p* values are displayed with different line colors based on 999 permutations. The pairwise correlations of these variables are presented in a color gradient representing the Spearman correlation coefficient. Size of the squares indicate the magnitude of the correlation. TC, soil total organic C; TN, soil total organic N; TP, soil total organic P; TK, soil total organic K; SCN, soil C:N ratio; SCP, soil C:P ratio; SNP, soil N:P ratio; AD, average degree; APD, average path distance; Cenbet, betweenness centrality; Cendeg, degree centrality; Ceneig, eigenvector centrality; Den, graph density; Trans, network transitivity.

Mantel test analysis was used to elucidate the underlying mechanisms responsible for the observed alterations in bacterial beta diversity. Results showed that ecological assembly processes and sub-network topological features of bacteria were significantly related to their beta diversity (Figure 5). For native communities, total dissimilarity and its replacement component were significantly correlated with community assembly processes, especially homogenizing dispersal (Mantel r ≥ 0.4, *p* < 0.01), and network topological features (Mantel r = 0.2–0.4, *p* < 0.01) including degree centrality, graph density, and network transitivity; nestedness dissimilarity was significantly correlated with the heterogeneous selection process for community assembly (Mantel r < 0.2, *p* < 0.05) and network features average distance (Mantel r < 0.2, *p* < 0.01; Figure 5b). However, the correlations of heterogeneous selection with both beta diversity and its components were not significant for invasive communities (*p* > 0.05). The correlation of homogenizing dispersal with BetaSOR becomes weaker (Mantel r = 0.2–0.4, *p* < 0.01), while the correlation of network characteristics with BetaSNE becomes stronger during invasion (Mantel r ≥ 0.4, *p* < 0.01; Figure 5a). These results confirmed that microbial community assembly and biotic interactions collectively determine soil bacterial composition, in addition to soil physiochemical properties. 

**Figure 6:**
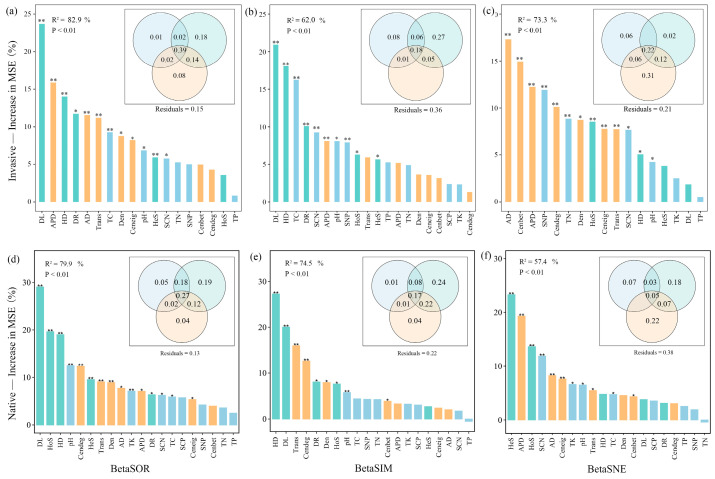
Variable importance in predicting bacterial beta diversity (**a**,**d**), its replacement (**b**,**e**) and nestedness (**c**,**f**) components for invasive (**a**-**c**) and native (**d**–**f**) communities based on a random forest prediction model computed as percentage of increase in mean squared error (MSE%). Significance of the models and R^2^ values were evaluated with 999 permutations of the response variables. Asterisks on columns indicate significance of explanatory variable on beta diversity with ** *p* < 0.01, * *p* < 0.05. The Venn plots nested within random forest plots depict variance partitioning of three groups of predictors, assembly process, network properties, and soil characteristics. The overlap represents shared variation among explanatory matrices. Numbers indicate adjusted R^2^. TC, soil total organic C; TN, soil total organic N; TP, soil total organic P; TK, soil total organic K; SCN, soil C:N ratio; SCP, soil C:P ratio; SNP, soil N:P ratio; AD, average degree; APD, average path distance; Cenbet, betweenness centrality; Cendeg, degree centrality; Ceneig, eigenvector centrality; Den, graph density; Trans, network transitivity.

Consistent with the Mantel test, variance partitioning and random forest analysis indicated that soil nutrients, community assemblage processes, and co-occurrence patterns determine the soil bacteria community composition of exotic and native plants along the elevation gradient (Figure 6). Community assembly and network topological features played greater roles than edaphic predictors (Figure 6). The contribution of ecological assemblage increased but network topology decreased for species replacement during invasion along altitudinal gradients (Figure 6b,e). In contrast, for species loss/gain during invasion, the importance of ecological assemblage decreased but network topology increased (Figure 6c,f).

## 4. Discussion

### 4.1. Beta Diversity Patterns along Altitude Gradient during Invasion

The variations in microbial community composition can be attributed to two opposing processes of turnover (species replacement) and nestedness (species loss). Our study demonstrated that bacterial community dissimilarities decreased during invasion (Figure 1, Table S1), and species replacement processes (91.4–91.6%) explained a substantially larger proportion than nestedness processes (8.4–8.6%; Table S1). Typically, high nestedness coincided with high variations in species richness, which inevitably led to diversity hotspots, and making low abundance communities a subset [24]. In contrast, small variations in species richness left little room for nestedness configurations and, therefore, there was lower nestedness. This observation parallels the low nestedness observed in temperate forests across northern China, where relatively minor variations in the species richness of large and small trees were noted [25]. Alternatively, nestedness emerges from habitat filtering across environmental gradients [26], whereby species that occur in harsh environments are a nested subset of species that occur in more benign environments [27]. It is generally accepted that native plants have developed broader ecological adaptations to their specific habitats over millions of years through co-evolution, whereas alien plants have been introduced and colonized in a relatively short timeframe, lacking adequate time to fully acclimate to surroundings and achieve their full potential altitude range [3]. Thus, exotic communities would be expected to exhibit more pronounced nestedness along elevational gradients in comparison to native communities. However, our results do not support this inference, as there is minimal difference in the nestedness components of exotic versus native communities. This result may emphasize the fact that bacterial community compositional variations of invasive plants along the elevation gradients were not the result of progressive filtering of the species pools at low elevation. Rather, invasive communities exhibit similar fitness to altitudinal gradients as native individuals.

The elevational transect has frequently been proposed as a valuable model for examining larger-scale ecological patterns, as it effectively condenses significant climatic variations within relatively narrow distances. Given that climate variability is assumed to increase with altitude, beta diversity could be inferred to decline at high altitude [28]. In our study, however, the beta diversity of soil bacterial community exhibited no significant decline patterns with increasing altitude, but rather a binominal pattern, which was inconsistent with hypothesis i (Figure 2). Similar results were observed in montane forests across China [29]. The possible explanation for this insignificant decay trend lies in that the assumption that climate is more temporally variable at high altitudes than at low altitudes may not be applicable. Despite the absence of direct climate measurement, a large-scale study on mountain climates across China previously provided evidence that the estimated annual ranges of temperature showed significant downward trends with elevation in the majority of cases [29]. In situ climatic measurement along the southern and northern slopes of Mt Taibai, which is the main peak of the Qinling Mountains, also showed a significant decline in the annual range of temperature along altitude, and no discernible trends were observed in the diurnal range of temperature [30]. Also, in South America, lower elevations to the west of the Andes have experienced the most substantial warming, whereas temperature increases at higher altitudes to the east have been more moderate [31].

Although there was an absence of decay patterns of bacterial composition community similarity in relation to geographic distance, significant decay patterns were observed in relation to environmental distance, especially soil pH (Figures S1–S3). The slope of this regression is the halving distance (i.e., the geographic distance needed to halve the initial similarity) [32], representing the turnover rates of species [33]. Microbial communities with higher turnover rates in their distance–decay relationships over environmental distance have been linked to decreased community stability [34]. Based on a steeper regression slope (Figures S1–S3), invasion is expected to increase the sensitivity of microbial communities to environmental change, which agrees with the higher heterogeneity that may occur in invasive habitats (Table S2). Zinger et al. [35] also observed the marine bacterial decay relationship to be steeper in ecosystems associated with high environmental heterogeneity, namely marine sediments and coastal environments, compared with pelagic ecosystems. In addition to soil pH, the importance of soil nutrient content and stoichiometry on bacterial community similarity was also discovered, indicating that substrate quality may affect the capacity of bacterial populations to adapt to invasive processes [36,37]. The lower quality of substrate, as indicated by the higher ratios of soil C:N (increased by 9.52%) and C:P (increased by 30.09%) (Table S2) after invasion limited the growth of soil bacterial, strengthened the competition for substrates, and thus reduced the community diversity. These observations indicate the weaker responsiveness of the community assemblage to the same degree of environmental changes during invasion, and environmental distances superseded the impacts of elevational distance on shaping the binominal pattern of bacterial community [38].

### 4.2. Assembly Process and Potential Effects on Bacterial Beta Diversity

In comparison to the community compositional nestedness component, the environmental distance–decay relationships were found to be stronger for species replacement, i.e., turnover (Figures S1–S3), which might mainly be the consequence of environmental sorting, or spatial and historical constraints [39]. To further identify ecological processes affecting bacterial composition (beta diversity), we conducted the iCAMP model. Results indicated that both deterministic and stochastic processes occur simultaneously in the assemblage of soil bacterial community along elevational gradients, while stochasticity plays more important roles, which was inconsistent with our hypothesis (ii). The potential explanation might be that the habitats are preferable for most soil microbes, and that release from environmental stress and weakened environmental filtering along elevational gradients resulted in a greater influence of stochastic (rather than deterministic) processes in structuring microbial communities [40]. This inference was supported by the increased importance of ecological drift, because drift is more important when selection is weak and the local community size is small [12]. Our results further revealed that the relative contribution of stochastic processes increased by 18.34% during invasion (Figure 3a). This is partly related to the higher competitive ability of invasive communities [41,42], which may contribute to environmental homogenization by simplifying plant–soil interactions and facilitating community convergence [17], where stochasticity may be critical in such relatively homogeneous ecosystems [43]. Conversely, the assembly of bacterial communities was found to be more deterministic for native communities. This implies that native bacterial communities may be more responsive to environmental fluctuations, potentially rendering them more susceptible to deterministic processes such as environmental filtration or biological interactions [44].

Stochastic processes, involved with ecological drift, dispersal limitation, and homogenizing dispersal, demonstrate an equal probability of dispersal for each species. The increased stochasticity in exotic plant communities is directly related to the increased contribution of dispersal limitation and ecological drift (Figure 3b). Dispersal limitation is a restriction on the ability of a species to disperse across habitats [45], and the influence of dispersal limitation on soil microorganisms is contingent upon the specific ecosystem or environmental habitat. The temperate biota, which have low UV, benign temperature, and better landscape connectivity, may be conducive to microbial community dispersal [38]. In contrast, the harsh environment of the alpine biota on the Tibetan Plateau, characterized by high UV, low temperature, and complex mountain terrain, would hinder the spatial dispersal of soil microbes [12,46]. Higher dispersal of soil bacterial communities in the rhizosphere of exotic plants suggests that the Qinling Mountains may be a suitable habitat for invasive communities and may further facilitate their spread. Furthermore, research has shown that ecological drift is more important in shaping soil microbial community composition under weak environmental selection [47].

Mantel analysis further revealed how the assembly mechanism led to differences in structural dissimilarity between exotic and native communities by altering different components of beta diversity. Specifically, stochastic processes affected the nestedness and turnover components of the bacterial beta diversity of invasive communities (Figure 5a), whereas only turnover components of native communities were affected (Figure 5b). This may indicate that the stronger effect of stochastic processes on species nestedness determines the lower variation in community composition in exotic communities, and ecological drift might be critical in this regard. The species dependence on the random placement influence on nestedness has also been reported by Wang et al. [25]. The nestedness indicates that lower richness communities are subsets of the higher richness communities, representing community compositional dissimilarities arising from non-random species loss [48]. Functional redundancy is essential for preserving community structure and ecological functionality in the face of potential species loss in the stressed environments along altitudinal gradients, through different species performing similar or identical functions [49]. Microorganisms are more susceptible to ecological drift in habitats with strong functional redundancy. The significant effect of stochastic processes on nestedness during invasion suggests that it is the increased ecological drift and functional redundancy that attenuates the effects of species loss on communities, manifested as lower dissimilarity in invaded communities. Interestingly, we also observed significant correlations between heterogeneous selection and nestedness dissimilarities. Most native plant species may initially establish themselves at low altitudes; however, as a result of the combined effects of heterogeneous selection pressure and worsening climatic conditions, coupled with a reduction in propagule pressure at higher elevations, these species are progressively eliminated from the lower altitudes and consequently diffused upward to higher altitudes [3,50]. Altogether, our results highlight that species loss or gain should also be considered as an important source of bacterial community dynamics during invasion, although the contribution of species nestedness in our study was lower than that found in other regions (usually <20%) around the world [51,52].

### 4.3. Potential Biotic Interaction and Effects on Bacterial Beta Diversity

The distribution of species is not solely contingent upon their capacity to survive in a suitable habitat; it also necessitates their ability to persist after establishment. Biological interactions, inferred from proportions of interacted and negative associations among microbial taxa, determine which species exist there and how they are organized as a community to persist [45]. The co-occurrence networks of native communities exhibited a greater prevalence of negative correlations than those of invasive species (Figure 5), suggesting higher antagonistic or competitive interactions for native communities. The availability of substrates represents a fundamental factor influencing the structures of soil microbial networks [53,54]; therefore, alterations in resource availability (e.g., organic carbon and organic nitrogen) through litter decomposition as well as root secretion from plant invasion [55,56] may represent a significant driver of these changes [57]. In addition, more competition among native species might lead to a lower efficiency of resource transfer compared with that of invasive communities, which could reduce the community stability. This may challenge the concept of complexity leading to stability [58,59], as the topological parameters of native co-occurrence networks showed significantly higher complexity (graph density, betweenness centrality, and network transitivity; Table S2).

Co-occurrence patterns have previously been identified as a deterministic process that regulates the intensity of intra-species and inter-species competition, generates niche partitioning, and limits community similarity, resulting in high beta diversity [19,60]. Nevertheless, we proved direct significant correlations between the topology of the co-occurrence network and the beta diversity of soil bacterial communities (Figure 5 and Figure 6). Previous research on soil potential diazotrophic beta diversity across the Tibetan Plateau also supports the idea that co-occurrence patterns have a greater effect on beta diversity than community assembly [61]. The biotic mechanisms underlying differences in beta diversity are generally considered to be related to ecological niche differentiation, i.e., resource competition largely drives community diversification, especially when microbial taxa have similar resource requirements or niches in a resource-poor environment [62]. The nutrient deficiency in native communities intensified inter-species resource competition, which has been postulated to constrain the coexistence of species and has ramifications for microbial community composition. However, we found that the correlation between beta diversity and biotic interactions was more pronounced in the invasion network, whereas in the native communities, there was only a weak correlation between several topological parameters (including cendeg, den, and trans) and beta diversity and its turnover component (Figure 5b). Random forests and variance decomposition analysis further indicated an increase in the contribution of co-occurrence patterns and a decrease in the contribution of assembly processes during invasion (Figure 6), consistent with our hypothesis (iii). One possible explanation for this result is that metabolic interdependence among taxa could also induce species coexistence that leads to the aggregation of microbes [63]. Invasion stimulated a great supply of soil-available nutrients, creating a more conducive environment for species to interact with each other and potentially enhancing the influence of biotic factors on community composition. Altogether, our results emphasize that species interdependence rather than competition is strongly correlated with bacterial biogeography during invasion, and that the inclusion of co-occurrence patterns as deterministic processes may be inappropriate.

## 5. Conclusions

This study identifies elevational patterns of bacterial beta diversity and associated microbial mechanisms in mountain ecosystems during plant invasion. The major conclusions are as follows. (1) The invasion of plants results in a reduction in beta diversity, mainly attributed to declined species turnover rather than species loss/gain. Inconsistent with hypothesis i, the community compositional dissimilarities exhibited a binominal pattern across the altitudinal gradient. This was likely due to decreased disparities in environmental heterogeneity over elevational distance as indicated by the robust decay correlation between beta diversity and environmental distances. The observed steeper slope of the environment distance–decay relationship in invasive plants indicated higher turnover rates, and, therefore, the greater responsiveness of invasive communities to environmental disturbance. (2) Inconsistent with hypothesis ii, stochastic processes played more important roles in the assemblage of the soil bacterial community, rather than deterministic processes. The effects of stochasticity were observed for the nestedness and turnover of invasive communities, but only for the turnover of native communities. This may indicate that the stronger effect of stochastic processes on species nestedness determines the difference in beta diversity between invasive and native communities, and ecological drift might be critical in this regard. (3) The correlation between beta diversity and biotic interactions was more pronounced in the invasion network since invasion stimulated a great supply of soil-available nutrients, creating a more conducive environment for species to interact with each other and potentially enhancing the influence of biotic factors on community composition. Consistent with hypothesis iii, the relative importance of network topology increased, while community assembly decreased, during the invasion process. In summary, these findings challenge the well-accepted geographical distance–decay relationships and provide a comprehensive understanding of the elevational patterns of beta diversity from a species replacement and loss/gain perspective in relation to community assembly and biotic interactions.

## Data Availability

The raw data supporting the conclusions of this article will be made available by the authors on request.

## Supplementary Materials

The following supporting information can be downloaded at: https://www.mdpi.com/article/10.3390/microorganisms12101972/s1, Figure S1: Relationships between total bacterial compositional dissimilarities and environmental gradients; Figure S2: Relationships between the turnover component of Sørensen dissimilarity (Simpson dissimilarities) and environmental gradients; Figure S3: Relationships between nestedness component of Sørensen dissimilarity and environmental gradients; Table S1: Comparisons of beta diversity between native and invasive communities among all samples; Table S2: Comparison of edaphic properties and network parameters between invasive and native communities.

## References

[1] Shi H., Zhou Q., Xie F.L., He N.J., He R., Zhang K.R., Zhang Q.F., Dang H.S. (2020). Disparity in elevational shifts of upper species limits in response to recent climate warming in the Qinling Mountains, North-central China. Sci. Total Environ..

[2] Zhou Y.J., Jia X., Han L., Tian G., Kang S.Z., Zhao Y.H. (2021). Spatial characteristics of the dominant fungi and their driving factors in forest soils in the Qinling Mountains, China. Catena.

[3] Zhang W.X., Yin D., Huang D.Z., Du N., Liu J., Guo W.H., Wang R.Q. (2015). Altitudinal patterns illustrate the invasion mechanisms of alien plants in temperate mountain forests of northern China. For. Ecol. Manag..

[4] Zhang G.L., Bai J.H., Tebbe C., Huang L.B., Jia J., Wang W., Wang X., Yu L., Zhao Q.Q. (2022). Plant invasion reconstructs soil microbial assembly and functionality in coastal salt marshes. Mol. Ecol..

[5] Custer G.F., van Diepen L.T. (2020). Plant invasion has limited impact on soil microbial α-diversity: A meta-analysis. Diversity.

[6] Tian C., Wang W.Q., Wang H.J., Chen H., Tian J.Q. (2023). Plant invasion mediates the regulation of topsoil organic carbon sequestration by the fungal community in coastal wetlands. Catena.

[7] Gong X., Chen Y.J., Wang T., Jiang X.F., Hu X.K., Feng J.M. (2020). Double-edged effects of climate change on plant invasions: Ecological niche modeling global distributions of two invasive alien plants. Sci. Total Environ..

[8] Ding Z., Liang J., Yang L., Wei C., Hu H., Si X. (2024). Deterministic processes drive turnover-dominated beta diversity of breeding birds along the central Himalayan elevation gradient. Avian Res..

[9] Wang J.J., Legendre P., Soininen J., Yeh C.F., Graham E., Stegen J., Casamayor E., Zhou J.Z., Shen J., Pan F.Y. (2020). Temperature drives local contributions to beta diversity in mountain streams: Stochastic and deterministic processes. Glob. Ecol. Biogeogr..

[10] Zheng Y., Ji N.N., Wu B., Wang J.T., Hu H.W., Guo L.D., He J.Z. (2020). Climatic factors have unexpectedly strong impacts on soil bacterial β-diversity in 12 forest ecosystems. Soil Biol. Biochem..

[11] Sardans J., Bartrons M., Margalef O., Gargallo-Garriga A., Janssens I., Ciais P., Obersteiner M., Sigurdsson B., Chen H., Peñuelas J. (2017). Plant invasion is associated with higher plant–soil nutrient concentrations in nutrient-poor environments. Glob. Change Biol..

[12] Zhou J., Ning D. (2017). Stochastic community assembly: Does it matter in microbial ecology?. Microbiol. Mol. Biol. Rev..

[13] Bell J.K., Siciliano S.D., Lamb E.G. (2023). Seasonality and bacterial community assembly processes dominate prairie ecosystem service disruption during invasion. Soil Biol. Biochem..

[14] Aslani F., Geisen S., Ning D., Tedersoo L., Bahram M. (2022). Towards revealing the global diversity and community assembly of soil eukaryotes. Ecol. Lett..

[15] Fan Q.P., Liu K.F., Wang Z.L., Liu D., Li T., Hou H.Y., Zhang Z.J., Chen D.H., Zhang S., Yu A.L. (2024). Soil microbial subcommunity assembly mechanisms are highly variable and intimately linked to their ecological and functional traits. Mol. Ecol..

[16] Liu L., Wang N., Liu M., Guo Z.X., Shi S.H. (2023). Assembly processes underlying bacterial community differentiation among geographically close mangrove forests. mLife.

[17] Chen X., Li H., Condron L.M., Dundield K.E., Wakelin S.A., Mitter E.K., Jiang N. (2023). Long-term afforestation enhances stochastic processes of bacterial community assembly in a temperate grassland. Geoderma.

[18] Xu Z.Y., Sun R.H., He T.Y., Sun Y.Z., Wu M.C., Xue Y.H., Meng F.Q., Wang J. (2023). Disentangling the impact of straw incorporation on soil microbial communities: Enhanced network complexity and ecological stochasticity. Sci. Total Environ..

[19] Xu L., He N.P., Li X.Z., Cao H.L., Li C.N., Wang R.L., Wang C.H., Yao M.J., Zhou S.G., Wang J.M. (2021). Local community assembly processes shape β-diversity of soil phoD-harbouring communities in the Northern Hemisphere steppes. Glob. Ecol. Biogeogr..

[20] Li W.J., Xia Y., Li N., Chang J., Liu J., Wang P., He X.W. (2024). Temporal assembly patterns of microbial communities in three parallel bioreactors treating low-concentration coking wastewater with differing carbon source concentrations. J. Environ. Sci..

[21] Liu C.X., Zhou Y., Qin H., Liang C.F., Shao S., Fuhrmann J.J., Chen J.H., Xu Q.F. (2021). Moso bamboo invasion has contrasting effects on soil bacterial and fungal abundances, co-occurrence networks and their associations with enzyme activities in three broadleaved forests across subtropical China. For. Ecol. Manag..

[22] Zhang G.L., Bai J.H., Tebbe C., Huang L.B., Jia J., Wang W., Wang X., Yu L., Zhao Q.Q. (2021). Spartina alterniflora invasions reduce soil fungal diversity and simplify co-occurrence networks in a salt marsh ecosystem. Sci. Total Environ..

[23] Lyu J.L., Yue M., Mao Z.X., Wang Y.C. (2023). Study on Invasion Status and Risk Assessment of Alien Plants in Qinling Mountains. Ecol. Environ..

[24] Si X., Baselga A., Ding P., Machado R.B. (2015). Revealing beta-diversity patterns of breeding bird and lizard communities on inundated land-bridge islands by separating the turnover and nestedness components. PLoS ONE.

[25] Wang X., Wiegand T., Anderson-Teixeira K.J., Bourg N.A., Hao Z., Howe R., Jin G., Orwig D., Spasojevic M., Wang S. (2018). Ecological drivers of spatial community dissimilarity, species replacement and species nestedness across temperate forests. Glob. Ecol. Biogeogr..

[26] Greve M., Gremmen N.J.M., Gaston K.J., Chown S.L. (2005). Nestedness of Southern Ocean island biotas: Ecological perspectives on a biogeographical conundrum. J. Biogeogr..

[27] Chase J.M. (2007). Drought mediates the importance of stochastic community assembly. Proc. Natl. Acad. Sci. USA.

[28] Stevens G.C. (1992). The elevational gradient in altitudinal range: An extension of Rapoport’s latitudinal rule to altitude. Am. Nat..

[29] Tang Z.Y., Fang J.Y., Chi X.L., Feng J.M., Liu Y.N., Shen Z.H., Wang X.P., Wang Z.H., Wu X.P., Zheng C.Y. (2012). Patterns of plant beta-diversity along elevational and latitudinal gradients in mountain forests of China. Ecography.

[30] Tang Z.Y., Fang J.Y. (2006). Temperature variation along the northern and southern slopes of Mt. Taibai, China. Agric. For. Meteorol..

[31] Vuille M., Bradley R.S., Werner M., Keimig F. (2003). 20th century climate change in the tropical Andes: Observations and model results. Climate Variability and Change in High Elevation Regions: Past, Present & Future.

[32] Baselga A. (2007). Disentangling distance decay of similarity from richness gradients: Response to Soininen et al. 2007. Ecography.

[33] Baselga A., Gómez-Rodríguez C. (2021). Assessing the equilibrium between assemblage composition and climate: A directional distance-decay approach. J. Anim. Ecol..

[34] Wu M.H., Chen S.Y., Chen J.W., Xue K., Chen S.L., Wang X.M., Kang S., Rui J., Thies J., Bardgett R. (2021). Reduced microbial stability in the active layer is associated with carbon loss under alpine permafrost degradation. Proc. Natl. Acad. Sci. USA.

[35] Zinger L., Boetius A., Ramette A. (2014). Bacterial taxa–area and distance–decay relationships in marine environments. Mol. Ecol..

[36] Stanek M., Zubek S., Stefanowicz A.M. (2021). Differences in phenolics produced by invasive Quercus rubra and native plant communities induced changes in soil microbial properties and enzymatic activity. For. Ecol. Manag..

[37] Xu Z.W., Guo X., Caplan J., Li M.Y., Guo W.H. (2021). Novel plant-soil feedbacks drive adaption of invasive plants to soil legacies of native plants under nitrogen deposition. Plant Soil.

[38] Zhang B., Xue K., Zhou S.T., Che R.X., Du J.Q., Tang L., Pang Z., Wang F., Wang D., Cui X.Y. (2022). Environmental selection overturns the decay relationship of soil prokaryotic community over geographic distance across grassland biotas. Elife.

[39] Villa P.M., Martins S.V., DinizÉcio S., de Oliveira-Neto S.N., Neri A.V., Pinto-Junior H., Nunes J.A., Bueno M.L., Ali A. (2021). Taxonomic and functional beta diversity of woody communities along Amazon forest succession: The relative importance of stand age, soil properties and spatial factor. For. Ecol. Manag..

[40] Li S., Li Y., Hu C., Zheng X., Zhang J., Zhang H., Bai N., Zhang H., Tian M., Ban S. (2021). Stochastic processes drive bacterial and fungal community assembly in sustainable intensive agricultural soils of Shanghai, China. Sci. Total Environ..

[41] Fahey C., Koyama A., Antunes P.M., Dunfield K., Flory S.L. (2020). Plant communities mediate the interactive effects of invasion and drought on soil microbial communities. ISME J..

[42] Shen K.P., Cornelissen H., Wang Y.J., Wu C.B., He Y.J., Ou J., Tan Q.Y., Xia T.T., Kang L.L., Guo Y. (2020). AM fungi alleviate phosphorus limitation and enhance nutrient competitiveness of invasive plants via mycorrhizal networks in karst areas. Front. Ecol. Evol..

[43] Luan L., Liang C., Chen L.J., Wang H.T., Xu Q.S., Jiang Y.J., Sun B. (2020). Coupling bacterial community assembly to microbial metabolism across soil profiles. Msystems.

[44] Huo X., Ren C.J., Wang D.X., Wu R.Q., Wang Y.S., Li Z.F., Huang D.C., Qi H.Y. (2023). Microbial community assembly and its influencing factors of secondary forests in Qinling Mountains. Soil Biol. Biochem..

[45] Wang Y.S., Li C.N., Tu B., Kou Y.P., Li X.Z. (2021). Species pool and local ecological assembly processes shape the β-diversity of diazotrophs in grassland soils. Soil Biol. Biochem..

[46] Zhang B., Xue K., Zhou S.T., Chen R.X., Du J.Q., Tang L., Pang Z., Wang F., Wang D., Cui X.Y. (2019). Phosphorus mediates soil prokaryote distribution pattern along a small-scale elevation gradient in Noijin Kangsang Peak, Tibetan Plateau. FEMS Microbiol. Ecol..

[47] Evans S., Martiny J.B., Allison S.D. (2017). Effects of dispersal and selection on stochastic assembly in microbial communities. ISME J..

[48] Liu W.X., Yang X., Jaing L., Guo L.L., Chen Y.R., Yang S., Liu L.L. (2022). Partitioning of beta-diversity reveals distinct assembly mechanisms of plant and soil microbial communities in response to nitrogen enrichment. Ecol. Evol..

[49] Fetzer I., Johst K., Schäwe R., Banitz T., Harms H., Chatzinotas A. (2015). The extent of functional redundancy changes as species’ roles shift in different environments. Proc. Natl. Acad. Sci. USA.

[50] Marini L., Bertolli A., Bona E., Federici G., Martini F., Prosser F., Bommarco R. (2013). Beta-diversity patterns elucidate mechanisms of alien plant invasion in mountains. Glob. Ecol. Biogeogr..

[51] Bertuzzi T., Marques Pires M., Maltchik L. (2019). Drivers of the beta diversity of aquatic plant communities along a latitudinal gradient in southern Brazilian coastal ponds. J. Veg. Sci..

[52] Fu H., Yuan G.X., Jeppesen E., Ge D.B., Li W., Zou D.S., Huang Z.R., Wu A.P., Liu Q.L. (2019). Local and regional drivers of turnover and nestedness components of species and functional beta diversity in lake macrophyte communities in China. Sci. Total Environ..

[53] de Camargo N.F., de Oliveira H.F.M., Ribeiro J.F., de Camargo A.J.A., Vieira E.M. (2019). Availability of food resources and habitat structure shape the individual-resource network of a Neotropical marsupial. Ecol. Evol..

[54] Lin Q., Li L., Adams J., Heděnec P., Tu B., Li C., Li T., Li X. (2021). Nutrient resource availability mediates niche differentiation and temporal co-occurrence of soil bacterial communities. Appl. Soil Ecol..

[55] Fahey C., Flory S.L. (2022). Soil microbes alter competition between native and invasive plants. J. Ecol..

[56] Zhang X., van Kleunen M., Chang C.L., Liu Y.J. (2023). Soil microbes mediate the effects of resource variability on plant invasion. Ecology.

[57] Wayne W., Schrama M. (2016). Identifying the role of soil microbes in plant invasions. J. Ecol..

[58] Qiu L.P., Zhang Q., Zhu H.S., Reich P.B., Banerjee S., Heijden M., Sadowsky M.J., Ishii S., Jia X.X., Shao M.A. (2021). Erosion reduces soil microbial diversity, network complexity and multifunctionality. ISME J..

[59] Xu M., Li X.L., Kuyper T., Xu M., Li X.L., Zhang J.L. (2021). High microbial diversity stabilizes the responses of soil organic carbon decomposition to warming in the subsoil on the Tibetan Plateau. Glob. Change Biol..

[60] Huang Y., Zhang X., Fu S., Zhang W. (2019). Environmental filtering drives local soil fungal beta diversity more than dispersal limitation in six forest types along a latitudinal gradient in Eastern China. Forests.

[61] Lei S.L., Wang X.T., Wang J., Zhang L., Liao L.R., Liu G.B., Wang G.L., Song Z.L., Zhang C. (2024). Effect of aridity on the β-diversity of alpine soil potential diazotrophs: Insights into community assembly and co-occurrence patterns. Msystems.

[62] Lee H., Bloxham B., Gore J. (2023). Resource competition can explain simplicity in microbial community assembly. Proc. Natl. Acad. Sci. USA.

[63] Jiao S., Chu H.Y., Zhang B.G., Wei X.R., Chen W.M., Wei G.H. (2022). Linking soil fungi to bacterial community assembly in arid ecosystems. iMeta.

