# Supplementary material for: Biotic Interactions Shape Soil Bacterial Beta Diversity Patterns along an Altitudinal Gradient during Invasion
Source: Microorganisms. 2024 Sep 28;12(10):1972. doi: 10.3390/microorganisms12101972 (PMC11509125; doi:10.3390/microorganisms12101972)
Supplement: Supplementary file 1 [file microorganisms-12-01972-s001.zip › microorganisms-3202228-supplementary.pdf]

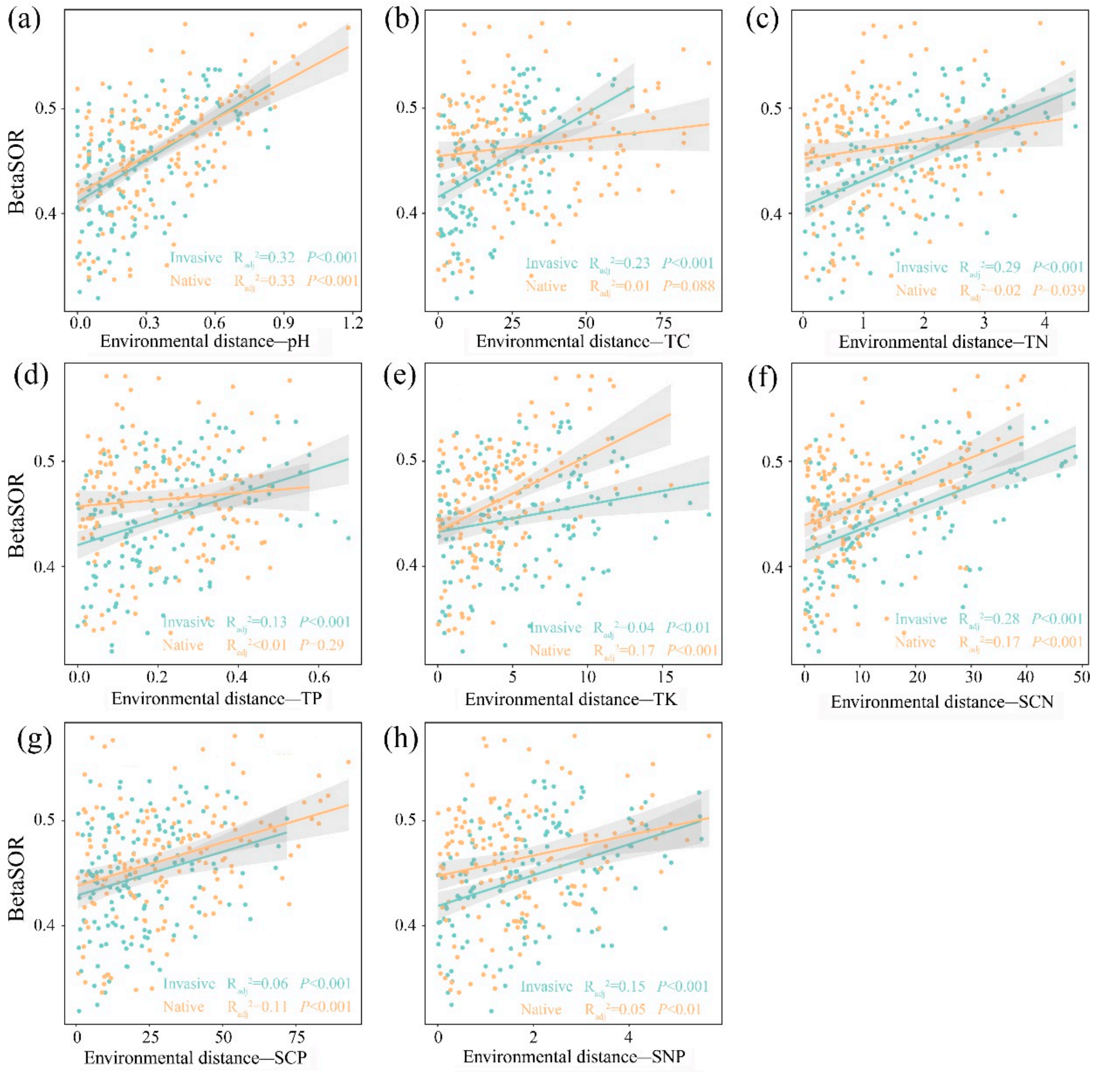

Figure S1 Relationships between total bacterial compositional dissimilarities and environmental gradients. The fitted linear models are shown as solid lines, with shading representing 95% confidence intervals. TC, soil total organic C; TN, soil total organic N; TP, soil total organic P; TK, soil total organic K; SCN, soil C:N ratio; SCP, soil C:P ratio; SNP, soil N:P ratio.

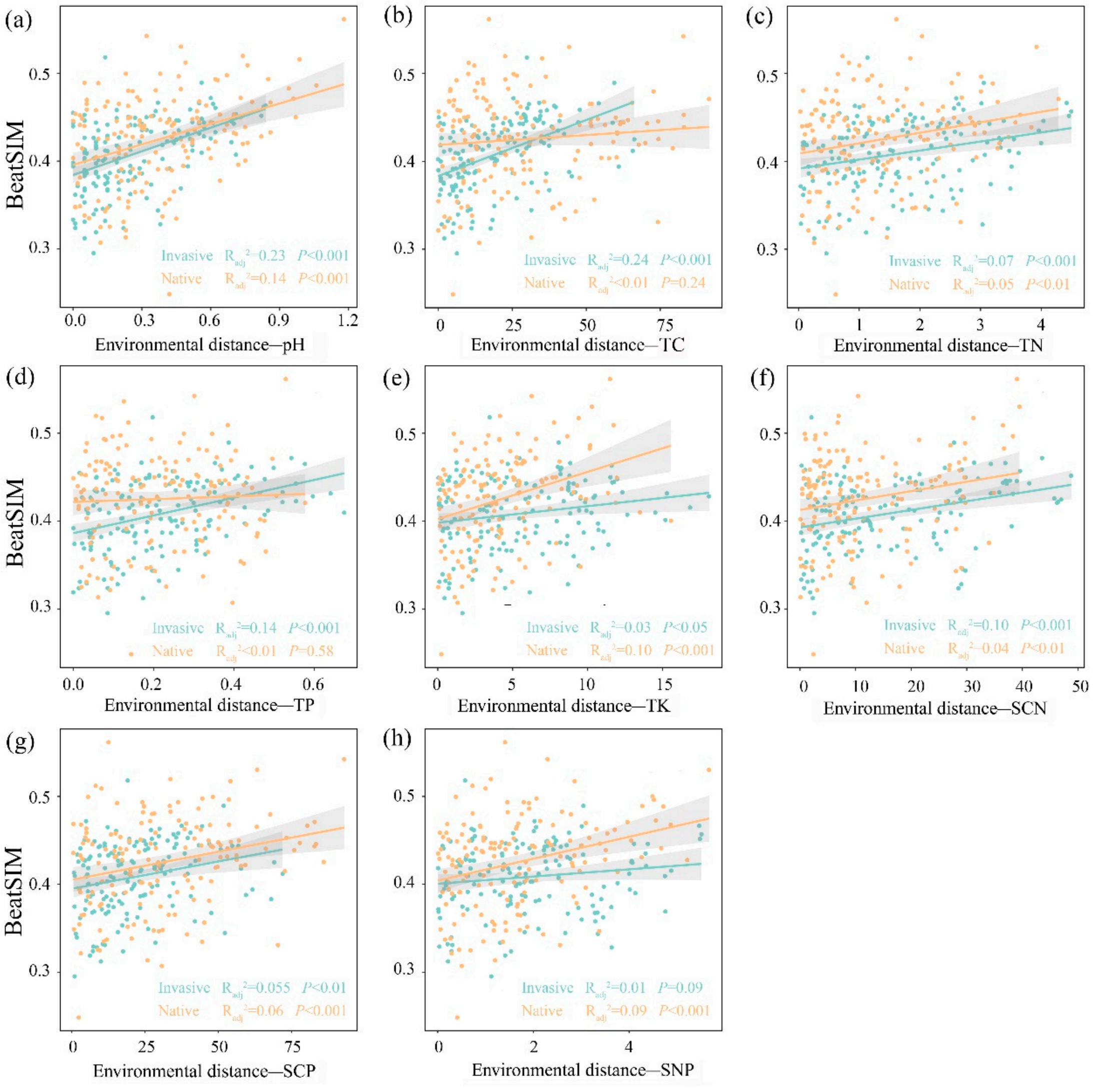

Figure S2 Relationships between the turnover component of Sørensen dissimilarity (Simpson dissimilarities) and environmental gradients. The fitted linear models are shown as solid lines, with shading representing 95% confidence intervals. TC, soil total organic C; TN, soil total organic N; TP, soil total organic P; TK, soil total organic K; SCN, soil C:N ratio; SCP, soil C:P ratio; SNP, soil N:P ratio.

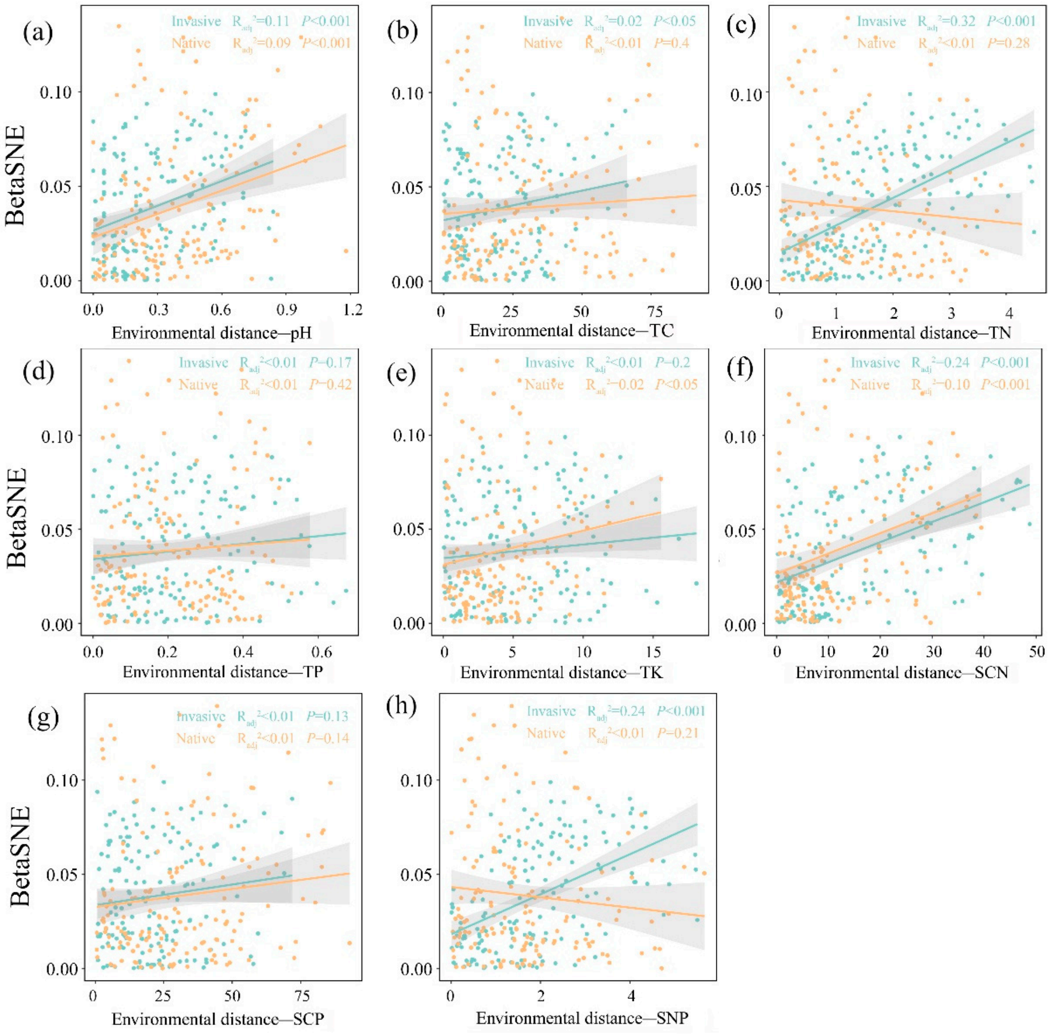

Figure S3 Relationships between nestedness component of Sørensen dissimilarity and environmental gradients. The fitted linear models are shown as solid lines, with shading representing 95% confidence intervals. TC, soil total organic C; TN, soil total organic N; TP, soil total organic P; TK, soil total organic K; SCN, soil C:N ratio; SCP, soil C:P ratio; SNP, soil N:P ratio.

Table S1 Comparisons of β diversity between native and invasive communities among all samples

	BetaSOR	BetaSIM	BetaSNE	BetaSIM/ BetaSOR	BetaSNE / BetaSOR
Invasive	0.223	0.204	0.019	0.914	0.086
Native	0.232	0.213	0.019	0.916	0.084

Table S2 Comparison of edaphic properties and network parameters between invasive and native communities

	Invasive	Native	F value	P value
pH	7.5722±0.3808	7.6816±0.3243	1.195	0.278
TC	37.737±18.377	38.8454±25.832	0.04	0.843
TN	2.0672±1.3816	1.886±1.20676	0.244	0.623
TP	0.83923±0.2474	0.85948±0.1831	0.102	0.751
TK	15.696±4.504	17.667±3.74444	2.792	0.0992
SCN	27.148±28.295	23.360±10.986	0.305	0.583
SCP	50.433±44.9127	32.856±26.923	0.414	0.522
SNP	2.5603±1.972	2.2245±1.495	0.436	0.511
AD	20.9680±3.186	26.015±2.3188	38.25	<0.001
APD	3.4184±0.087	3.4866±0.0382	10.24	0.00207
Cenbet	0.0238±0.00345	0.0141±0.0025	118.8	<0.001
Cendeg	0.1285±0.0154	0.1024±0.0093	45.77	<0.001
Ceneig	0.9011±0.0086	0.8911±0.0047	21.7	<0.001
Den	0.02084±0.0024	0.0234±0.0015	17.21	<0.001
Trans	0.3663±0.0184	0.4167±0.0223	90.78	<0.001

TC, soil total organic C; TN, soil total organic N; TP, soil total organic P; TK, soil total organic K; SCN, soil C:N ratio; SCP, soil C:P ratio; SNP, soil N:P ratio; AD, average degree; APD, average path distance; Cenbet, betweenness centrality; Cendeg, degree centrality; Ceneig, eigenvector centrality; Den, graph density; Trans, network transitivity.
